# Proactive Locomotor Adjustments Are Specific to Perturbation Uncertainty in Below-Knee Prosthesis Users

**DOI:** 10.1038/s41598-018-20207-5

**Published:** 2018-01-30

**Authors:** Matthew J. Major, Chelsi K. Serba, Xinlin Chen, Nicholas Reimold, Franklyn Ndubuisi-Obi, Keith E. Gordon

**Affiliations:** 1 Northwestern University Department of Physical Medicine and Rehabilitation, Chicago, United States; 2 Jesse Brown VA Medical Center, Chicago, United States; 3 Northwestern University Department of Physical Therapy and Human Movement Sciences, Chicago, United States; 4 Edward Hines, Jr. VA Hospital, Hines, United States

## Abstract

Sensory-motor deficits associated with below-knee amputation impair reactions to external perturbations. As such, below-knee prosthesis users rely on proactive control strategies to maintain locomotor stability. However, there are trade-offs (metabolic, comfort, etc.) associated with proactive strategies. We hypothesize that because proactive control strategies are costly, prosthesis users and non-impaired participants will use *a priori* knowledge (timing, direction) of an impending lateral perturbation to make specific gait adaptations only when the timing of the perturbation is known and the adaptation can be temporally-limited. This hypothesis was partially supported. When the perturbation timing was predictable, only prosthesis users, and only on their impaired side, increased their lateral margin of stability during the steps immediately preceding the perturbation when perturbation direction was either unknown or known to be directed towards their impaired side. This strategy should reduce the likelihood of requiring a corrective step to maintain stability. However, neither group exhibited substantial proactive adaptations compared to baseline walking when perturbation timing was unpredictable, independent of perturbation direction knowledge. The absence of further proactive stabilization behaviors observed in prosthesis users in anticipation of a certain but temporally unpredictable perturbation may be partially responsible for impaired balance control.

## Introduction

People use a combination of proactive and reactive control strategies to maintain locomotor stability^1^. Proactive control occurs prior to a perturbation, and includes both sensory-based anticipation (e.g. visual observation of a hazard) and, a learned predictive strategy to adapt locomotor patterns in preparation for the perturbation^1^. Reactive control occurs after the perturbation. This feedback-driven response is important for reacting to unanticipated perturbations and for correcting errors^2^. The contribution of each control strategy to maintain locomotor stability is dependent on many factors including: ability to anticipate^3^ and sense^2^ a perturbation, prior experience^4^, and control strategy costs (metabolic^5^, cognitive^6^, etc.), and risk assessment^7^.

Approximately half of community-living persons with lower limb amputation fall each year^8^. For below-knee prosthesis users (BKPUs), the loss of proprioceptive feedback and active ankle joint control may impair reactive control strategies^9–13^. These deficits may particularly challenge the maintenance of frontal-plane locomotor stability^14,15^, as frontal-plane stability depends heavily on active control in comparison to sagittal plane stability that benefits from passive dynamics^16–18^. As such, BKPUs often exhibit proactive control strategies to increase frontal-plane stability including; slow walking velocities, selecting faster, shorter, and wider steps, and greater lateral margins of stability (MoS) than non-impaired counterparts^19–21^.

Curiously, BKPUs appear to make only small anticipatory gait adaptations when repeatedly exposed to discrete but temporally-unpredictable lateral perturbations^22^. This result is surprising considering that BKPUs are both fall prone^8^ and able to adapt gait patterns to further increase frontal-plane stability as evidenced by their response to environments that continuously challenge mediolateral balance^15,20,23,24^. However, it is reasonable that BKPUs may choose to make minimal proactive adaptations to increase stability in preparation for discrete perturbations because evidence suggests that such adaptations are costly, requiring metabolic energy^5^, limiting maneuverability^25,26^, and potentially increasing discomfort due to soft tissue stress placed on the residuum^27^. Accordingly, when BKPUs anticipate a temporally-unpredictable perturbation with a known recoverable magnitude (i.e., below the threshold which would result in a fall) as employed in the study by Sturdy *et al*.^22^, choosing to maintain their preferred (already cautious) gait pattern would help avoid those costs associated with further proactive adaptations to increase stability. Consequently, the provision of spatiotemporal knowledge of a perturbation could in theory minimize the negative trade-offs associated with proactive gait stability strategies by encouraging context-specific adaptations. For example, knowing the timing of a perturbation during continuous walking would allow proactive strategies to be temporally-restricted to only the steps immediately preceding the perturbation rather than incurring an ongoing cost every step. Moreover, knowledge of both timing and direction could then encourage even more focused adaptions to prepare for an impending perturbation.

Therefore, the purpose of this study was to quantify the effects of *a priori* spatiotemporal information of a discrete, lateral perturbation on proactive locomotor strategies employed by non-impaired controls and BKPUs walking with passive prosthetic components (i.e., no active joint control). Given the theorized relationship between preferred control strategies and contextual information of a recoverable perturbation as driven by locomotor costs (metabolic, maneuverability), we aimed to address two primary hypotheses: H1) when perturbation instance is unknown and there is an inability to limit anticipatory costs, neither group would exhibit proactive gait adaptations irrespective of perturbation direction knowledge compared to baseline walking; and H2) when perturbation instance is known and anticipatory costs could be limited to a finite number of steps, both groups would exhibit time-dependent proactive strategies to enhance stability just prior to the perturbation onset. As a secondary hypothesis (H2b), we expected that when timing and direction of the perturbation was known, proactive adaptations of BKPUs would be more focused and asymmetric to specifically increase stability on the side of impaired limb due to the loss of sensory mechanisms and active joint control. Ultimately, our results from studying the effects of contextual perturbation information on proactive strategies of BKPUs and their differences compared to non-impaired individuals would enhance understanding of the mechanisms used by BKPUs to maintain locomotor stability.

## Methods

### Participants

Northwestern University Institutional Review Board approved the protocol, with the methods carried out in accordance with the relevant guidelines and regulations, and participants provided written informed consent including use of video and images expressing their likeness. All participants met the following inclusion criteria: 18 to 65 years, normal or corrected vision, and able to walk 10 minutes continuously without undue fatigue or health risks. BKPUs met additional inclusion criteria: unilateral below-knee amputation, daily use of their clinically-prescribed prosthesis for ambulation without a mobility aid, at least one year experience using a prosthesis, and a residuum in good condition (no scars, ulcers, infections, etc.). Exclusion criteria included: musculoskeletal (apart from amputation) and/or vestibular pathologies affecting balance, currently on medications affecting proprioception and/or balance, and cognitive deficits that preclude understanding of testing instructions.

### Experimental setup

**Figure 1:**
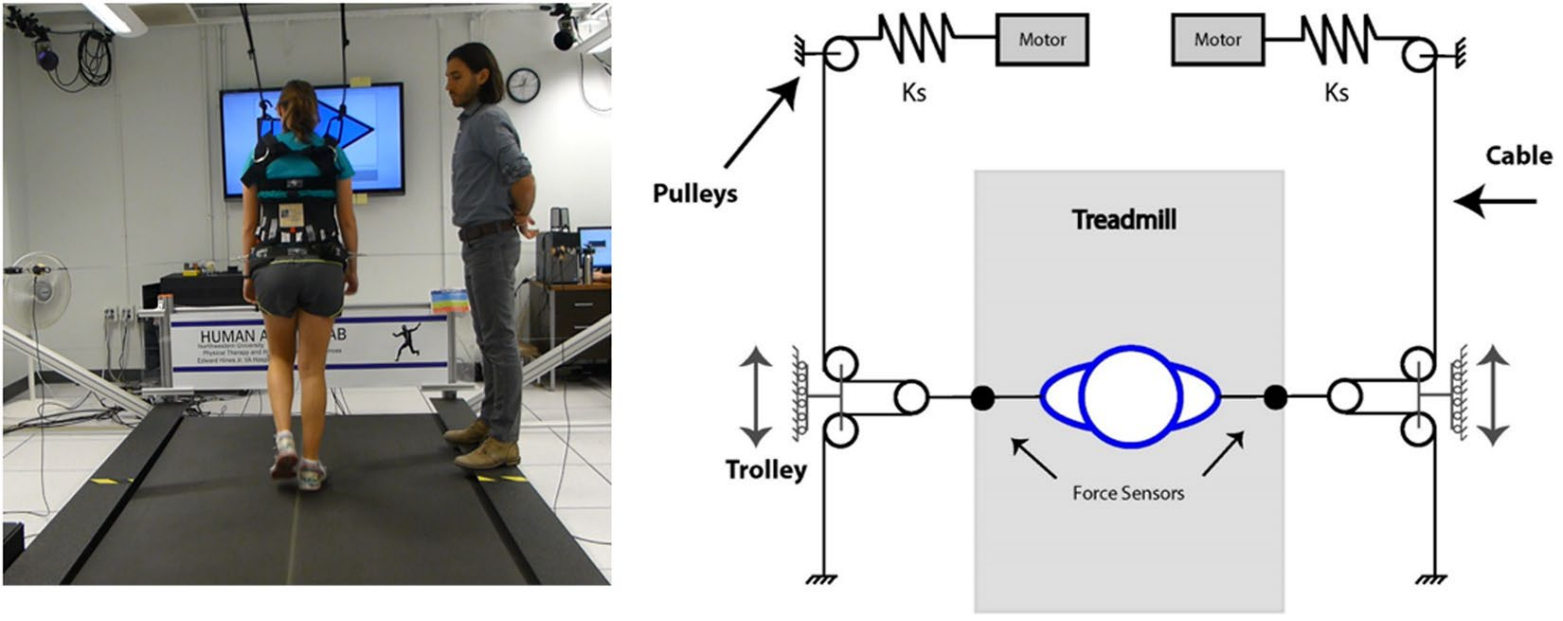
A participant-perspective image (left) and top-down schematic (right) of the perturbation robot.

Participants walked on an oversized treadmill, belt width 1.39 m (Tuff Tread, Willis, TX), providing space to respond to lateral perturbations. During walking, participants wore a trunk harness attached to a passive overhead safety device (Aretech, Ashburn, VA) that provided no bodyweight support and did not restrict frontal-plane motion (see Supplementary Data videos S1 and S2). Lateral perturbations were applied during walking at random times throughout the gait cycle using a custom-built, cable robotic device^28,29^ (Fig. 1). Participants wore a pelvis harness attached to a pair of cables. Independent series-elastic linear motors created force on each cable. Load cells measured the applied forces. During all walking the cable-robot operated in transparent mode – zero net lateral force applied to the participant through equal and opposite tensile forces – to maintain continuous tension in the system. Lateral perturbations were created by modulating the load in each cable-motor such that a net lateral force of 12% bodyweight was applied for 400 msec. At the completion of a perturbation, the system returned to transparent mode operation. Based on pilot testing, we selected this perturbation magnitude as it was both challenging and recoverable (did not cause a fall or require participants to stop walking) for BKPUs.

A 60-inch monitor mounted 1.8 m in front of the treadmill and audio speakers provided visual and auditory feedback about the direction and timing of upcoming lateral perturbations (Fig. 1).

Retro-reflective markers were attached to the pelvis (superior iliac crests, anterior-superior iliac spines, spine sacral level 2, and two tracking markers) and bilaterally on the greater trochanter, lateral knee, lateral malleolus, calcaneus, and second and fifth metatarsals. Prosthetic foot markers were positioned to approximately match the sound foot. A ten-camera motion capture system recorded 3-D marker positions at 100 Hz (Qualisys, Göteborg, Sweden).

### Experimental protocol

First, we recorded participant demographics and additional information from BKPUs on prosthesis use (type, experience), time since amputation, amputation etiology, functional balance, and socket comfort. Functional balance was measured using the Berg Balance Scale, known to be valid and reliable for lower limb prosthesis users^30^. The comfort of the prosthetic socket was evaluated with the Socket Comfort Score, a common ordinal-scale clinical outcome measure^31^.

Participants’ preferred self-selected walking speed was then identified through a staircase method of increasing and decreasing the treadmill speed until desired speed was confirmed through verbal feedback. Participants were then given treadmill walking practice to familiarize them with the setup and protocol which lasted on average two minutes.

Following these initial assessments, participants then performed a series of walking trials at their preferred speed that were grouped into blocks of perturbation condition based on provided *a priori* knowledge:

1. Baseline
2. Unknown Time and Direction
3. Unknown Time and Known Direction
4. Known Time and Direction
5. Known Time and Unknown Direction

Each of these blocks consisted of six trials, and as all blocks were repeated twice except for Baseline to collect an equal number of perturbations directed to the right and left side, this resulted in each participant completing a total of 54 trials. The testing order was randomized to minimize order bias although learning effects remain a possibility within each testing block. Conditions 2 and 3 were used to address the first primary hypothesis (H1), while conditions 4 and 5 addressed our second primary (H2) and secondary hypotheses (H2b). For the Known Direction conditions 3 and 4, all perturbations in a block were in the same direction. For the Unknown Direction conditions 2 and 5, perturbation direction was randomized within the block. Participants experienced the audio-visual feedback and the associated lateral perturbation (or lack thereof) for each condition during quiet standing immediately prior to beginning each testing block so that they would be able to anticipate the perturbation type and magnitude. Additionally, participants were informed that they would either not experience a perturbation (Baseline) or would experience a perturbation with *a priori* knowledge of the perturbation direction and/or timing prior to beginning each testing block.

Knowledge of the perturbation direction was provided using the monitor to display an arrow indicating the perturbation direction (left or right). A question mark was displayed when perturbation direction knowledge was unknown. Knowledge of the perturbation timing was provided using a progress bar timer (a visual rectangle that would fill from left to right - fully filled at the instant of perturbation) and a synchronized audible 5-second countdown. During conditions when perturbation timing knowledge was unknown, the rectangle would not fill and no audio countdown was provided. During Baseline trials, although no perturbation was provided, a question mark was displayed on the monitor and participants received both visual and audio timing feedback.

Participants were instructed to walk along the center of the treadmill, marked by a distinct yellow chalk line, and to return to the treadmill center as quickly as possible following perturbations. Participants were specifically told not to treat the chalk line as a “tight-rope.” Participants were informed that rest between trials and testing blocks were permitted for as long as requested to minimize fatigue bias. Although participants regularly utilized rest breaks between blocks, none requested breaks between trials and so each block was tested during continuous walking. Subsequent trials (each discrete perturbation) were only started when the participant was observed to have fully regained steady-state walking (approximately 20 steps) and the treadmill belt was gradually stopped after each block.

### Data Analysis

Qualisys processing software was used to fill gaps in tracked marker positions. Visual3D (C-Motion, Germantown, MD) software was used to low-pass filter marker position trajectories (Butterworth, 6 Hz cut-off frequency). A Visual3D pelvis model was created using the 7 pelvis markers and instantaneous mediolateral whole-body center-of-mass (BCoM) position was estimated as the center of this model.

Initial contact and toe-off for each limb were estimated in Visual3D software as the maximum fore-aft distance between the calcaneus and pelvis center, and minimum distance between the 5^th^ metatarsal and pelvis center, respectively. All of the event times were manually (visually) confirmed for accuracy and adjusted as appropriate. Custom MATLAB (Mathworks, Natick, MA) software was used to estimate the lateral margin-of-stability (MoS), step width, step length, and step time for each limb. Step width and MoS were assessed given their suggested relationship with generalized frontal-plane balance control^32^, while step length and time provide insight into gait regulation and can also affect frontal-plane stability^33^. Specifically, MoS was selected as means to quantify frontal-plane control as it not only demonstrates relationships with fall risk^34–36^, but is also an intuitive biomechanical outcome permitting step-by-step analysis^37^ which was necessary to address our time-dependent hypothesis (H2). There exist other outcome measures such as nonlinear dynamic metrics which are also related to dynamic stability and falls but require data from several continuous minutes of walking^32^ and were hence not ideal for this analysis. Furthermore, although our approximation of BCoM position was based on a simplified pelvis model, evidence suggests that this technique is comparable to other more complex multi-segment models for estimation of MoS^37^.

Step width and length for a given limb and step were defined as the medial-lateral and anterior-posterior distance, respectively, between the heel markers of contralateral limb at initial contact and the following initial contact of limb in question. Step time was defined as the time between initial contact events, and stance time for a given limb was defined as the time between its initial contact and toe-off. MoS was defined as the minimum distance between the instantaneous lateral extrapolated BCoM position, a velocity weighted lateral BCoM (*BCoM*_*Lat*_) position, and the 5^th^ metatarsal marker (*BoS*_*R/L*_) lateral position during stance^33^. Specifically, the MoS was calculated as:

1 $$Bo{S}_{R/L}-\,(BCo{M}_{Lat}+VBCo{M}_{Lat}/\sqrt{\frac{g}{l}})$$

where, *R/L* refers to the right or left stance limb, *VBCoM*_*Lat*_ is lateral velocity of the *BCoM*_*Lat*_, *g* is the acceleration due to gravity (9.81 m/s^2^), and *l* is approximated as trochanter height (m) times 1.34 (effective pendulum length for the inverted pendulum model). Based on this equation, all MoS values were positive as the extrapolated BCoM position remained within the boundaries of the metatarsal positions.

For the Unknown Time and Baseline conditions, these parameters were averaged separately for each limb across trials and the four steps preceding (*n-1* through *n-4*) the perturbation step (*n*), which was defined as the step with the last initial contact prior to the perturbation instance. For the Known Time conditions, parameters were averaged across trials and separately for the first (*n-1* and *n-2*) and second (*n-3* and *n-4*) steps preceding the perturbation step (*n*). To ensure that equal number of trials were analyzed for each condition, only the first block of trials involving perturbations of an Unknown Direction were used for analysis and this choice had the advantage of also minimizing undesired effects of learning bias.

### Statistical analysis

To address our hypotheses, separate three-way mixed ANOVAs (one between-group factor, two within-groups factors) were used to test main and interaction effects on MoS, step width, step length, and step time for data corresponding to either the Unknown (H1) or Known Time (H2) conditions. For the Unknown Time conditions, the tested main effects were group (BKPU, control), limb (impaired/sound, non-dominant/dominant), and perturbation direction (baseline, toward impaired/non-dominant limb, toward sound/dominant limb, and unknown). For the Known Time conditions, the tested main effects were group (BKPU, control), limb (impaired/sound, non-dominant/dominant), and step (baseline, 1^st^ step before perturbation step (step n-1 or 2), and 2^nd^ step before perturbation (n-3 or 4)). The Known Time analysis was conducted separately for each perturbation direction (toward impaired/non-dominant limb, toward sound/dominant limb, and unknown) to address our secondary hypothesis (H2b) of the effects of perturbation direction knowledge. Baseline data were included in both ANOVA models as representative data when participants did not expect or experience a perturbation. Prior to final analysis and interpretation, an absence of violations of normality were confirmed using the Shapiro-Wilk test, and violations of sphericity were assessed using the Mauchly’s test. If the assumption of sphericity was violated, results were interpreted using a Greenhouse-Geisser correction. Bonferroni adjustments were used for multiple *post hoc* pairwise comparisons to minimize risk of Type-I error.

The frequency of limb with initial contact and the support phase (single or double) just prior to the perturbation instance (i.e., step *n*) were also recorded for each condition as means to aid interpretation of stability measure results. A Freidman analysis was performed separately for each group to analyze the main effect of *a priori* knowledge on frequency (total number of trials) of the sound/dominant limb at initial contact and single support phase at step *n*. This analysis assessed if participants were more likely to have a particular limb in stance at the perturbation instance and if they were positioned in either single or double limb support.

All statistical analyses were conducted using SPSS software (v24, IBM, Armonk, NY). The critical alpha for all analyses was 0.05.

### Data availability

The datasets generated during and/or analyzed during the current study are available in the Northwestern University cloud-based digital repository, [https://digitalhub.northwestern.edu/collections/09e7110d-7677-4b82-985a-a7c26ac46b57].

## Results

Six BKPUs using their prescribed prostheses and 13 non-impaired controls participated in the study (Table 1). BKPU participants were experienced and regular prosthesis users (all reported use at 7 days/week), with comfortable sockets at testing time, and possessed high levels of functional balance (Table 1). All BKPUs walked with a non-articulated dynamic prosthetic foot. One BKPU participant was not comfortable walking with perturbations of 12% bodyweight. The perturbation magnitude was reduced to 10% bodyweight for all trials for this participant. These data were included in the analysis as the perturbations produced an observable destabilizing effect that was confirmed by the participant.

**Table 1:** Participant characteristics.

Controls (n = 13: 7 female/6 male)	*Age (years)*	29 (11.0)	Average (St. Dev)
	*Mass (kg)*	65.3 (9.7)	
	*Height (m)*	1.68 (0.07)	
Below-knee prosthesis users (n = 6: 5 female/1 male, 2 dysvascular amputation etiology/4 traumatic etiology)	*Age (years)*	48 (8)	
	*Mass (kg)*	70.2 (11.3)	
	*Height (m)*	1.65 (0.07)	
	*Time since amputation (years)*	14.0 (11.3–17.5)	Median (interquartile range)
	*Prosthesis use experience (years)*	13.0 (11.0–17.3)	
	*Prosthesis use frequency (hours/day)*	15.8 (12.9–16.0)	
	*Berg Balance Scale (ordinal scale 0–56)*	55 (54–56)	
	*Socket Comfort Score (ordinal scale 0–10)*	9 (8–10)	

The average walking speeds (±SD) for controls and BKPUs were 1.3 ± 0.1 m/s and 0.8 ± 0.3 m/s, respectively. All participants completed the full set of testing blocks. Although noticeably destabilized by the perturbations, all participants were able to successfully recover (i.e., no falls, and no stoppage or change in treadmill belt speed) without assistance using a combination of side and cross-over stepping strategies (see Supplementary Data videos S1 and S2). Rest periods between testing blocks were used by all participants.

**Figure 2:**
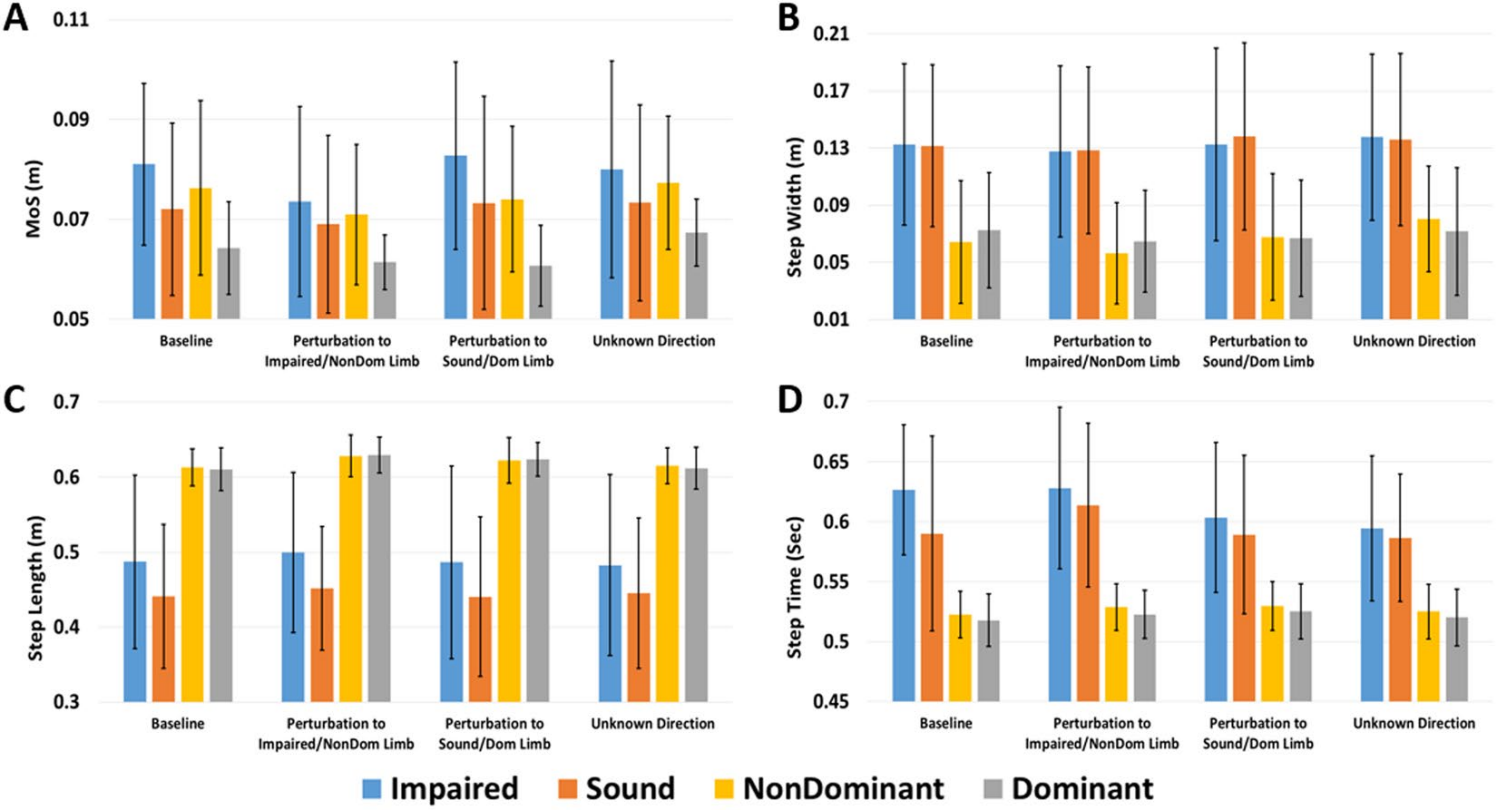
Average (**A**) MoS, (**B**) step width, (**C**) step length, and (**D**) step time across four steps preceding Unknown Time perturbation step for each side and condition. Error bars denote 95% CI.

**Figure 3:**
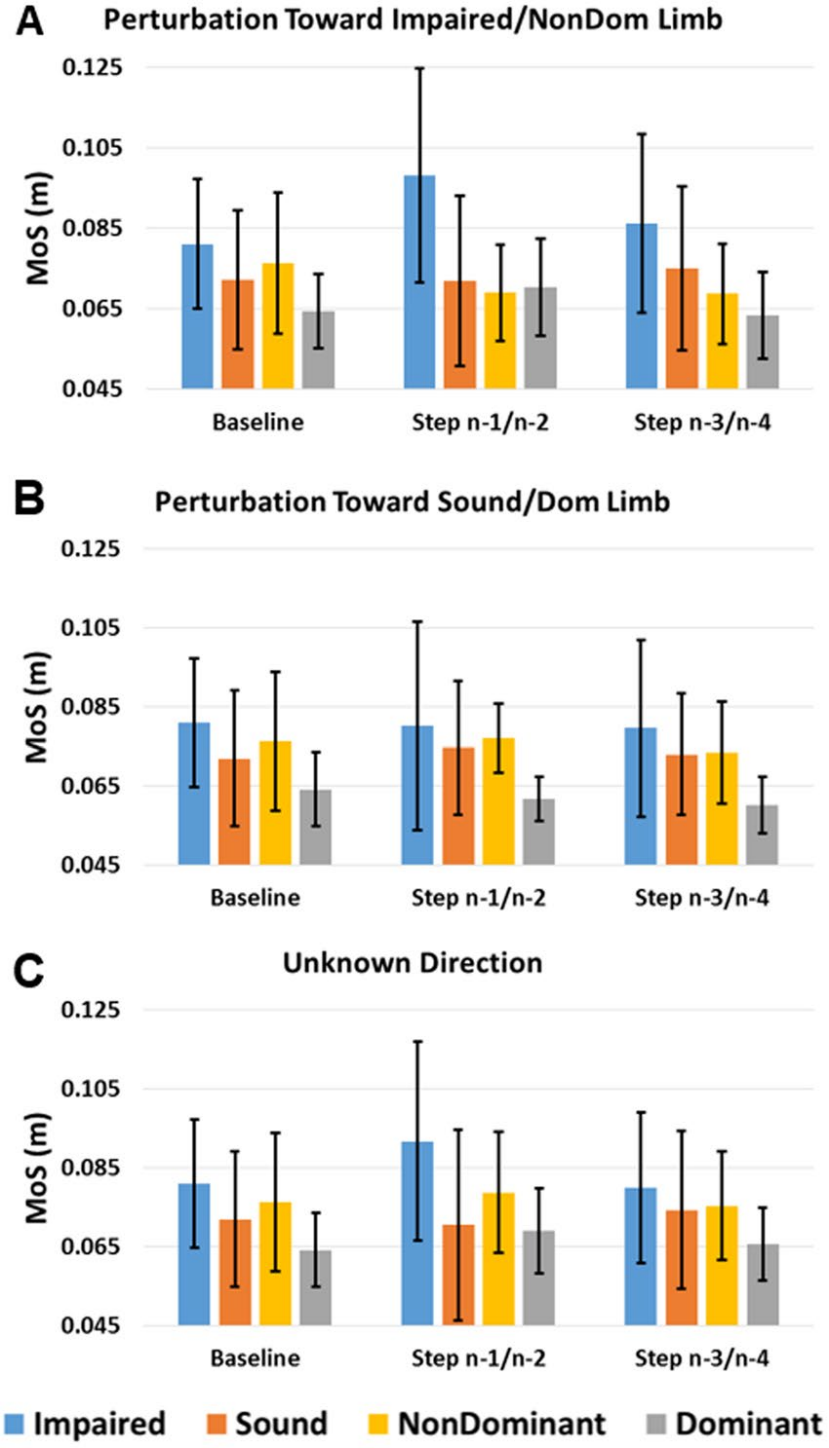
Average MoS for baseline and steps preceding perturbation step for each side during Known Time perturbation toward (**A**) impaired/non-dominant, (**B**) sound/dominant, and (**C**) unknown side. Error bars denote 95% CI.

**Figure 4:**
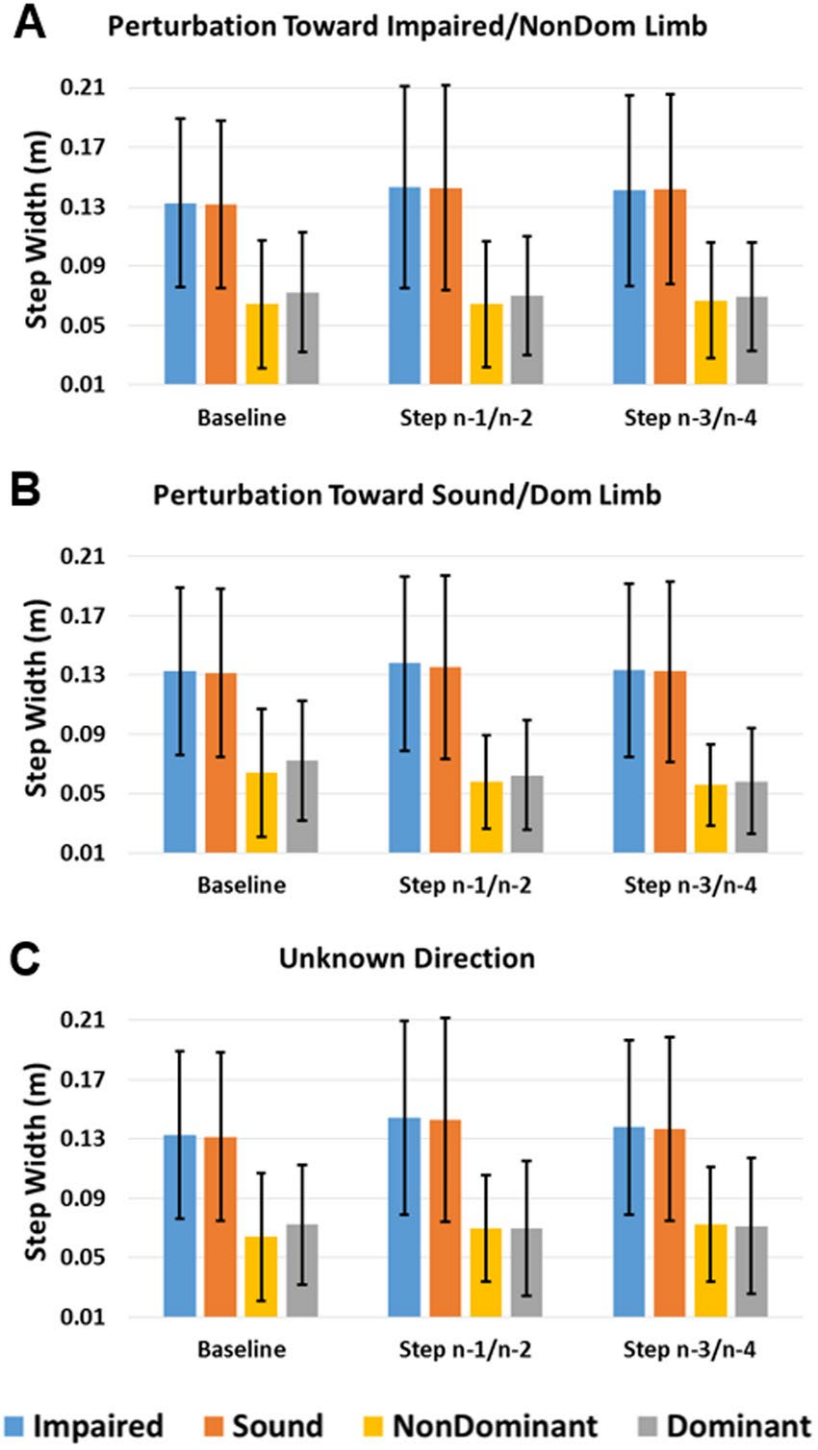
Average step width for baseline and steps preceding perturbation step for each side during Known Time perturbation toward (**A**) impaired/non-dominant, (**B**) sound/dominant, and (**C**) unknown side. Error bars denote 95% CI.

**Figure 5:**
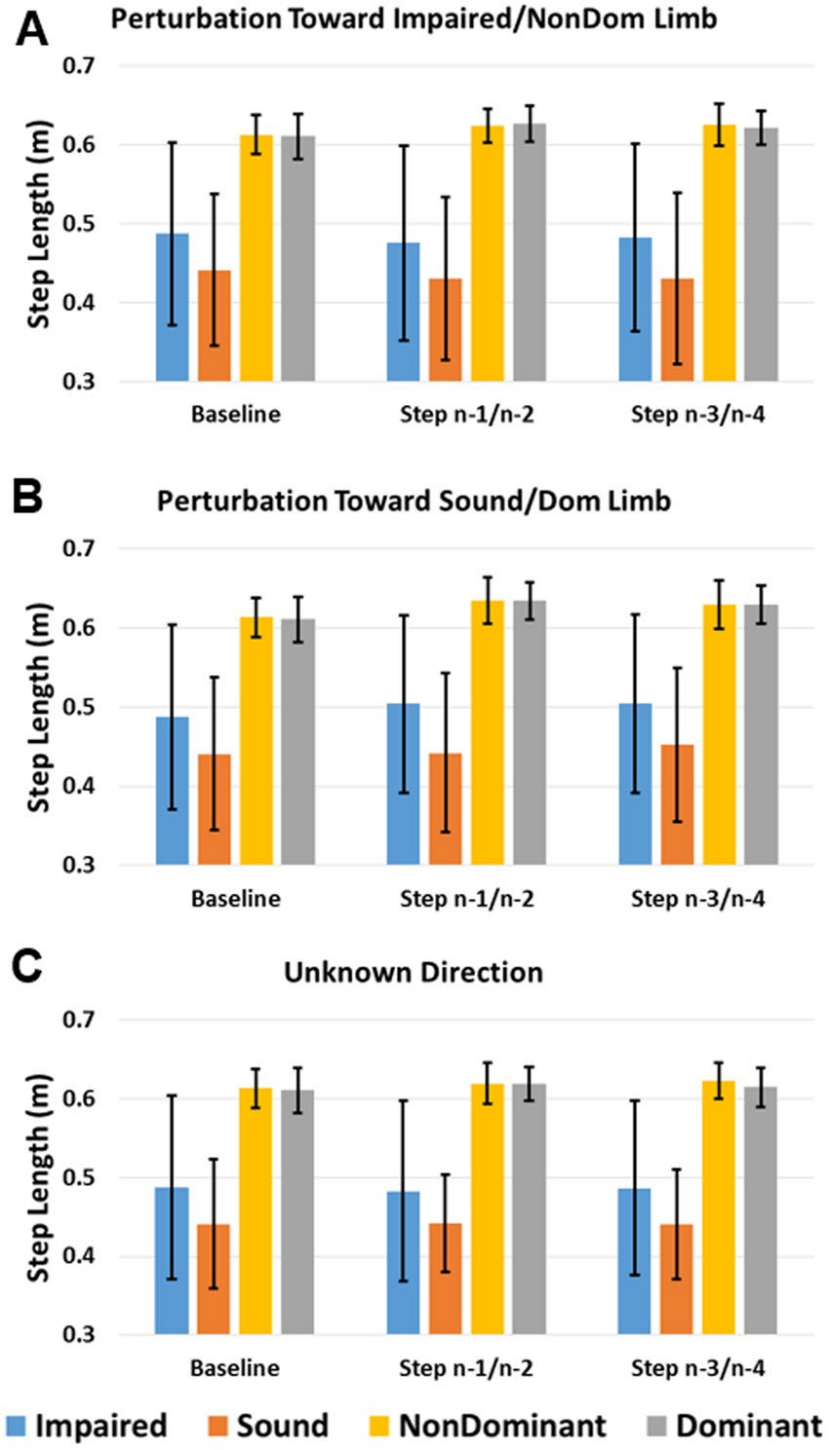
Average step length for baseline and steps preceding perturbation step for each side during Known Time perturbation toward (**A**) impaired/non-dominant, (**B**) sound/dominant, and (**C**) unknown side. Error bars denote 95% CI.

**Figure 6:**
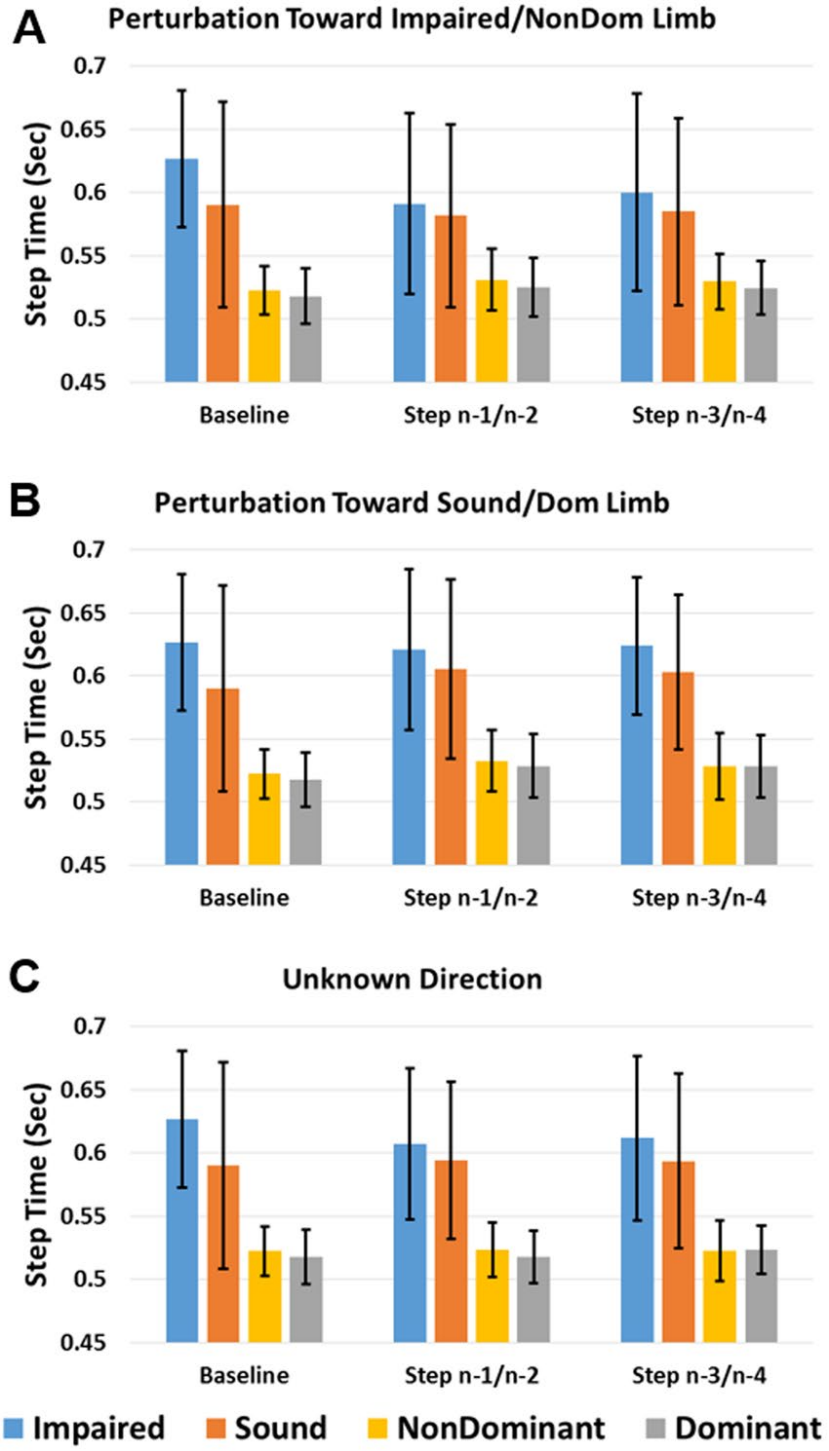
Average step time for baseline and steps preceding perturbation step for each side during known-time perturbation toward (**A**) impaired/non-dominant, (**B**) sound/dominant, and (**C**) unknown side. Error bars denote 95% CI.

**Figure 7:**
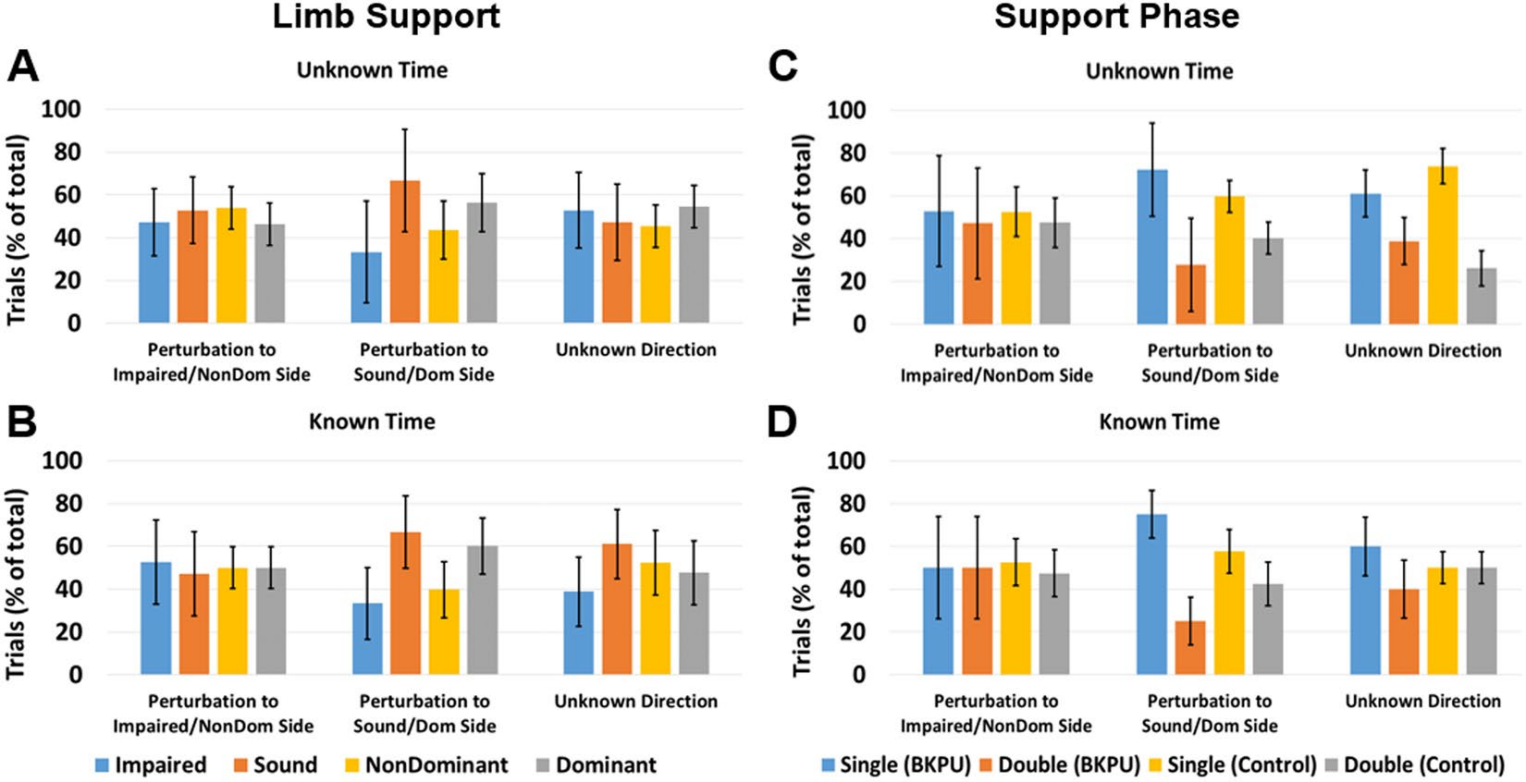
Frequency (percentage) of limb with initial contact just prior to (**A**) Unknown Time and (**B**) Known Time perturbation, and frequency of support phase (single or double) at time of (**C**) Unknown Time and (**D**) Known Time perturbation for each condition. Error bars denote 95% CI. *BKPU* and *Control* notation refer to the support phase of BKPUs and able-bodied controls, respectively.

Average MoS, step width, step length, and step time across subjects and four steps preceding the perturbation step for each side and direction of the Unknown Time perturbations are displayed in Fig. 2. These data are separated by individual steps for the Known Time conditions in Figs 3–6. The percentage frequency of last limb to contact the ground prior to perturbation (perturbation step) and the support phase (single limb or double limb) at the instant of perturbation for each condition are found in Fig. 7. For Figs 2–7, the impaired and sound limb refer to the BKPUs, while the dominant and non-dominant limb refer to the control group.

### Unknown Time

For MoS, the main effect of perturbation condition was significant (F(3,51) = 4.014, p = 0.012)(Fig. 2A), with a reduction in MoS across limbs and groups for perturbation toward the impaired/non-dominant limb compared to perturbation toward the sound/dominant limb (p = 0.047) and when direction is unknown (p = 0.009). Additionally, the main effect of limb was significant (F(1,17) = 5.171, p = 0.036), with MoS of the impaired/non-dominant limb greater than the sound/dominant limb. The main effect of group was not significant (F(1, 17) = 1.786, p = 0.199). For step width, the main effect of perturbation condition was significant (F(3, 51) = 3.101, p = 0.035), but *post hoc* comparisons were not significant (p ≥ 0.064) (Fig. 2B). The main effect of group also was not significant (p = 0.065).

For step length, the main effect of limb was significant (F(1,17) = 10.990, p = 0.004) with the impaired/non-dominant limb step length greater than the sound/dominant limb (Fig. 2C). Additionally, the interaction effect of limb*group was significant (F(1,17) = 13.593, p = 0.002), with differences in step length greater for the BKPU group compared to controls. The main effect of group on step length was also significant (F = (1,17) = 13.515, p = 0.002) with control group step length greater than the BKPU group.

For step time, the main effect of limb was significant (F(1,17) = 4.741, p = 0.044), with step time for the impaired/non-dominant limb greater than the sound/dominant limb (Fig. 2D). The main effect of perturbation condition was significant (F(2.327, 39.552) = 3.224, p = 0.044), but *post hoc* comparisons were not significant (p ≥ 0.108). However, the interaction effect of condition*group was significant (F(2.327, 39.552) = 3.352, p = 0.039), with step time differences between conditions greater for the BKPU group than the control group. Additionally, the main effect of group was significant (F(1,17) = 10.850, p = 0.004), with step time for the BKPU group greater than controls.

### Known Time and Known Perturbation Direction: Perturbation directed toward impaired/non-dominant limb

For MoS, the three-way interaction of step*limb*group was significant (F(2,34) = 5.468, p = 0.009) (Fig. 3A). The main effect of limb was also significant (F(1,17) = 6.615, p = 0.020) with the impaired/non-dominant limb MoS greater than the sound/dominant limb. Upon further inspection, the step*limb interaction was not significant for either group (p ≥ 0.077), but BKPUs more clearly demonstrated greater impaired limb MoS at the steps just prior to perturbation (n-1/2) compared to the second set of prior steps (n-3/4) and baseline. The main effect of group was not significant (F(1, 17) = 2.843, p = 0.110). For step width, no main or interaction effects were significant (p ≥ 0.061) (Fig. 4A).

For step length, the main effect of limb was significant (F(1,17) = 14.745, p = 0.001) with step lengths greater for the impaired/non-dominant limb compared to the sound/dominant limb (Fig. 5A). The interaction effect of limb*group was also significant (F(1,17) = 26.952, p < 0.001) with differences greater for the BKPU group than controls. The main effect of group was also significant (F(1,17) = 14.0.14, p = 0.002) with step length for the controls greater than the BKPU group.

For step time, the main effect of step was significant (F(1.354, 23.013) = 4.316, p = 0.039), but *post hoc* comparisons were not significant (p ≥ 0.053) (Fig. 6A). However, the interaction effect of step*group was significant (F(1.354, 23.013) = 7.680, p = 0.006) with differences greater for the BKPU group compared to controls. The main effect of limb was significant ((F(1, 17) = 6.994, p = 0.017) with step time for the impaired/non-dominant limb greater than sound/dominant limb. Additionally, the main effect of group was significant (F(1,17) = 6.807, p = 0.018) with step time greater for the BKPU group than control group.

### Known Time and Known Perturbation Direction: Perturbation directed toward sound/dominant limb

For MoS, only the main effect of limb was significant (F(1,17) = 5.162, p = 0.036), with the impaired/non-dominant limb MoS greater than the sound/dominant limb, while the main effect of group was not significant (F(1,17) = 2.051, p = 0.170) (Fig. 3B). For step width, only the main effect of group was significant (F(1,17) = 4.753, p = 0.044) with step widths greater for the BKPU group than controls (Fig. 4B).

For step length, the main effect of limb was significant (F(1,17) = 20.145, p < 0.001), with step lengths greater for the impaired/non-dominant limb compared to the sound/dominant limb (Fig. 5B). Additionally, the interaction effect of limb*group was significant (F(1,17) = 27.945, p < 0.001) with differences greater for the BKPU group than controls. The main effect of group was also significant (F(1,17) = 13.146, p = 0.002) with step length for the controls greater than the BKPU group.

For step time, the main effect of limb was significant (F(1,17) = 8.110, p = 0.011) with step times of the impaired/non-dominant limb greater than the sound/dominant limb (Fig. 6B). The interaction effect of limb*group was significant (F(1,17) = 6.474, p = 0.021) with differences greater for the BKPU group than controls. The main effect of group was also significant (F(1,17) = 12.278, p = 0.003) with step time for the BKPU group greater than controls.

### Known Time and Unknown Perturbation Direction

For MoS, the three-way interaction step*limb*group was significant ((F(2,34) = 5.345, p = 0.010). The main effect of limb was significant (F(1, 17) = 6.700, p = 0.019), with the impaired/non-dominant limb MoS greater than the sound/dominant limb (Fig. 3C). The interaction effect of step*limb was also significant across groups (F(2,34) = 5.607, p = 0.008), with differences between steps greater for the impaired/non-dominant limb compared to the sound/dominant limb. Specifically, the impaired/non-dominant limb exhibited the largest increase in MoS of the steps just prior to perturbation (n-1/2) compared to second set of prior steps (n-3/4) and baseline. Upon further inspection, this effect was primarily due to the BKPUs as the interaction effect of step*limb was only significant for this group (F(2,10) = 11.612, p = 0.002). The main effect of group was not significant (F(1,17) = 1.539, p = 0.232). For step width, no main or interactions effects were significant (p ≥ 0.055) (Fig. 4C).

For step length, the main effect of limb was significant (F(1,17) = 13.093, p = 0.002), with step lengths greater for the impaired/non-dominant limb compared to the sound/dominant limb (Fig. 5C). Additionally, the interaction effect of limb*group was significant (F(1,17) = 17.683, p = 0.001) with differences greater for the BKPU group than controls. The main effect of group was also significant (F(1,17) = 14.503, p = 0.001) with step length for the controls greater than the BKPU group.

For step time, the main effect of limb was significant (F(1,17) = 6.491, p = 0.021) with step times for the impaired/non-dominant limb greater than the sound/dominant limb (Fig. 6C). However, the interaction effect of limb*group was not significant (p = 0.055). The main effect of group was also significant (F(1,17) = 10.298, p = 0.005) with step time for the BKPU group greater than controls.

### Limb and Support Phase at the Perturbation Instant

The main effect of *a priori* knowledge on the last limb to contact the ground prior to the perturbation was not significant for either group (p ≥ 0.345) (Fig. 7A and B). The main effect of condition on support phase at the instant of perturbation was significant for only the control group (p = 0.023), but *post hoc* comparisons were not significant (p ≥ 0.059) (Fig. 7C and D).

## Discussion

This study explored the effects of *a priori* knowledge (timing and direction) of an impending lateral perturbation on the proactive locomotor strategies of BKPUs and compared these strategies to non-impaired control participants.

In support of our first primary hypothesis (H1), our findings suggest that without knowledge of the perturbation timing, BKPUs and controls do not modify their MoS irrespective of *a priori* information of the perturbation direction. Although the MoS across groups and limbs was smaller for perturbations toward the impaired/non-dominant limb, none of these conditions were different than Baseline. Worth noting is that across groups and conditions, a persistent asymmetry in MoS exists with bias toward greater MoS on the impaired or non-dominant limb. In anticipation of unknown time perturbations, BKPU participants did make small increases in step width and decreases in step time. Although small, these proactive modifications should theoretically enhance frontal-plane stability^5,33,38^ and are consistent with previous research findings that individuals with below-knee amputation make only minimal proactive gait adaptations in response to repeated mediolateral perturbations^22^. However, the absence of clear proactive adaptations is inconsistent with findings that non-impaired populations make proactive modifications in preparation for destabilizing events that will occur at an unknown time^39–41^, and that BKPUs increase step width while walking with continuous perturbations^20,23^, or over unstable terrain^19,24^. The strategy to minimally make proactive adaptions to further increase frontal-plane stability, as observed in this study, may be driven by the associated costs of changing gait for an indefinite time period to prepare for a discrete perturbation. For example, adapting wider steps will increase the metabolic cost of transport^5^, limit maneuverability^25^, and could potentially create discomfort due to moments applied at the distal end of the socket that ultimately generate unfavorable pressures applied to the residuum^42–44^. Furthermore, the discrete nature of the perturbation in this study contrasts the continuous disturbances employed in the other referenced BKPU studies^19,20,23,24^ and may partially explain this inconsistency in results.

Our second primary hypothesis (H2) that both participant groups would make time-dependent proactive gait adaptations preceding known time perturbations, and the accompanying secondary hypothesis (H2b) that these adaptations for BKPUs would be specific to perturbation direction knowledge were partially supported. Although both groups consistently exhibited greater MoS on the impaired or non-dominant limb during the Known Time conditions, controls demonstrated no evident proactive gait adaptations while BKPUs appeared to increase impaired limb MoS during the step just prior to perturbation when the perturbation direction was unknown or known to be directed towards the impaired limb (Fig. 3). At least for the condition of known perturbation to the impaired limb, the increase in MoS may be partially due to the observed quickening of steps^33^ (Fig. 6). Increasing MoS should result in a proportional increase in the lateral impulse required to move the XCoM beyond the base-of-support^45^. In theory, perturbations that do not result in the XCoM moving outside the base-of-support should passively self-stabilize, without requiring a corrective step^45^. As such, increasing lateral MoS of the impaired limb may serve to reduce the active control required to recover from the perturbation. Selecting proactive strategies that reduce the requirement to actively respond to perturbations could be desirable considering BKPU’s lost physiological (sensory and active joint) mechanisms^9,10,14,33^ that prohibit rapid stance-limb ankle torque center-of-pressure corrections and delay obstacle avoidance response times^12^. In addition, reducing the probability that a cross-over corrective step will be required is desirable as this action introduces a risk of the limbs colliding^46,47^. Interestingly, increasing MoS during the steps immediately prior to perturbation was not clearly observed in control participants or in BKPUs when the perturbation was known to be directed toward the sound limb. It is possible that a greater perturbation magnitude is required to generate sufficient risk to elicit proactive gait modification for discrete predictable perturbations directed toward an intact limb. It is important to recognize that while the step*limb interaction effect on MoS for BKPUs was significant for the condition of Known Time and Unknown Direction, only the three-way interaction was significant for the perturbation direction toward the impaired/non-dominant limb and the specific BKPU adaptation was confirmed through observation of trends. Therefore, this promising result in BKPU behavior and its clinical importance warrants further exploration.

Although not significant, BKPUs had a noticeable trend to vary the frequency of both support phase (single or double) and the last limb to contact the ground (impaired or sound) prior to the instance of perturbation (Fig. 7). Across both the Known and Unknown Time conditions, with knowledge that the perturbation would be directed toward the sound limb BKPUs were two times more likely to be on their sound limb than their impaired limb and three times more likely to be in single limb support than double support at the instant of perturbation. This bias is likely created by the observed asymmetries in step length and time (Figs 2C,D, 5 and 6). Consequently, it may be that the observed change when the perturbation direction was either unknown or known to be directed toward the impaired limb was intentional to increase the probability of having both limbs in contact with the ground at the instant of perturbation. Such an action would theoretically enhance frontal-plane stability by making both legs available to re-center BCoM position^48,49^, but given the non-significance of these results, this supposition warrants further investigation.

Several limitations should be considered when interpreting these results. First, the statistical power of this study was limited by the small sample size of BKPUs, but this study served as a means for identifying potentially important factors of BKPU locomotor stability for further investigation. Additionally, the convenience sample of BKPUs represents a group of relatively high-functioning, experienced prosthesis users. Future work should consider inclusion of a wider range of mobility levels to enhance external validity of such findings. Higher functioning prosthesis users were important for this study given the nature of the protocol as participants needed to be comfortable with the experimental tasks. Also, we did not vary perturbation magnitudes. Changing the intensity of the perturbation may change anticipatory behaviors due to a real or perceived increase in fall risk. The selected perturbation magnitude was sufficient to noticeably destabilize all participants while allowing them to successfully recover and return to steady-state locomotion. Finally, although testing blocks were randomized to account for order bias, there still remained a risk of learning effects within each testing block. Although test blocks were limited to six trials to minimize both fatigue and learning effects, the results should interpreted accordingly.

The clinical implications of these findings suggest that BKPUs implement specific proactive motor strategies when there is likelihood of a perturbation towards the impaired limb. Importantly, it appears that these preparatory changes are dependent on the *a priori* knowledge of the perturbation. Specifically, when a lateral perturbation can be anticipated but the exact timing cannot be predicted BKPUs make only small increases in step width and decreases in step time without noticeable changes in MoS. In contrast when the perturbation timing is known, BKPUs selected time-dependent proactive behaviors (e.g., increasing MoS just before perturbation instance). The implications of this finding is that contextual information of the environment is important to BKPUs for selecting proactive mechanisms to enhance frontal-plane stability and deemphasize the requirements to appropriately react to perturbations. The practical risk for not implementing clear proactive strategies unless perturbation timing is known is that BKPUs may not be optimally positioned for recovering from perturbations during ambulation. Although implementing proactive strategies for an indefinite period of time may be costly and hence avoided, the decision to select gait patterns that optimize for multiple factors including stability may explain the high prevalence of falls in this group. Future work should therefore focus on understanding the consequences of selecting these proactive strategies to perturbation recovery to better understand the mechanisms by which BKPUs fall and/or recover from perturbations. A greater understanding of stability control mechanisms could inform device and therapeutic interventions (i.e., training paradigms) for enhancing frontal-plane stability to ultimately improve ambulation safety^13,50^. Theoretically, quantifying how individual prosthesis users (or groups categorized by motor capacity) integrate sensory information and environmental cues to prepare for locomotor stability threats will assist with designing personalized rehabilitation interventions.

## Conclusions

The results from this study partially support our hypotheses. We observed that BKPUs and able-bodied controls minimally adapted proactive strategies to further enhance frontal-plane stability in anticipation of discrete perturbations occurring at an unpredictable time. When the timing of perturbation was predictable, BKPUs made proactive gait adjustments that were specific to *a priori* knowledge of the perturbation direction, i.e., increasing lateral MoS. In contrast, control participants did not demonstrate proactive gait adaptations in anticipation of known time perturbations. Additionally, the data suggest that BKPUs select slower walking speeds with shorter and slower steps independent of condition when compared to control participants. These general strategies that are present every step may be potential methods to enhance continuous frontal-plane stability.

## Electronic supplementary material

Supplement 1

Supplement 2
